# Multi-Level Comparative Framework Based on Gene Pair-Wise Expression Across Three Insulin Target Tissues for Type 2 Diabetes

**DOI:** 10.3389/fgene.2019.00252

**Published:** 2019-03-26

**Authors:** Shaoyan Sun, Fengnan Sun, Yong Wang

**Affiliations:** ^1^School of Mathematics and Statistics, Ludong University, Yantai, China; ^2^Clinical Laboratory, Yantaishan Hospital, Yantai, China; ^3^CEMS, NCMIS, MDIS, Academy of Mathematics and Systems Science, Chinese Academy of Sciences, Beijing, China; ^4^Center for Excellence in Animal Evolution and Genetics, Chinese Academy of Sciences, Kunming, China

**Keywords:** type 2 diabetes, gene pairwise expression, dysfunctional interactions, multi-level analysis, random walk with restart

## Abstract

Type 2 diabetes (T2D) is known as a disease caused by gene alterations characterized by insulin resistance, thus the insulin-responsive tissues are of great interest for T2D study. It’s of great relevance to systematically investigate commonalities and specificities of T2D among those tissues. Here we establish a multi-level comparative framework across three insulin target tissues (white adipose, skeletal muscle, and liver) to provide a better understanding of T2D. Starting from the ranks of gene expression, we constructed the ‘disease network’ through detecting diverse interactions to provide a well-characterization for disease affected tissues. Then, we applied random walk with restart algorithm to the disease network to prioritize its nodes and edges according to their association with T2D. Finally, we identified a merged core module by combining the clustering coefficient and Jaccard index, which can provide elaborate and visible illumination of the common and specific features for different tissues at network level. Taken together, our network-, gene-, and module-level characterization across different tissues of T2D hold the promise to provide a broader and deeper understanding for T2D mechanism.

## Introduction

Type 2 diabetes (T2D) is one of the leading complex diseases. It is most commonly seen in older adults, but it is increasingly seen in children, adolescents, and younger adults due to rising levels of obesity, physical inactivity, and poor diet (International Diabetes Federation [IDF], 2017). T2D is mainly a glucose metabolism disorder, and is currently believed to be a heterogeneous disease. One important fact is that the development of T2D involves multiple tissues (Zhong et al., 2010; Camastra et al., 2011; Petersen et al., 2012; Tang et al., 2018). Those tissues include white adipose tissue, skeletal muscle, and liver (shortly written as adipose, muscle, and liver hereafter). Each tissue has its own characteristics induced by T2D. One important and challenging problem is to explore the cross talk among multiple tissues since T2D is a systemic disease involving complicated synergy and regulation among different tissues. Exploring its underlying mechanisms across multiple tissues will be helpful for personalized therapy and precision medicine in T2D treatment. However, most existing studies focused on single tissue only (Li et al., 2015; Lee and Kim, 2016; Alghamdi et al., 2017).

On the other hand, it has been accepted that although molecules are basic components of cellular machinery, a complex disease is generally caused not from the malfunction of individual molecules but from the interplay of a group of correlated molecules or a network. Thus, with the development of bioinformatics and high-throughput data, network-based characterization of complex diseases, including T2D, has been invaluable to integrate and interpret functional genomics datasets and identify new biomarkers or modules to better classify patients into subtypes. Such approaches are much more powerful than approaches that examine a single gene at a time (Barabási et al., 2011; Hofree et al., 2013; Zhang et al., 2014; Liu et al., 2017; Zhang and Zhang, 2017; Hwang et al., 2018; Zou et al., 2018). However, most methods tended to characterize the complex programs using sets of genes, while the interaction (described as edge in biological network) information was not fully utilized in final prediction or analysis. Therefore, such methods cannot inform us on the functional discrepancies between gene pairs that are perturbed under disease.

**FIGURE 1:**
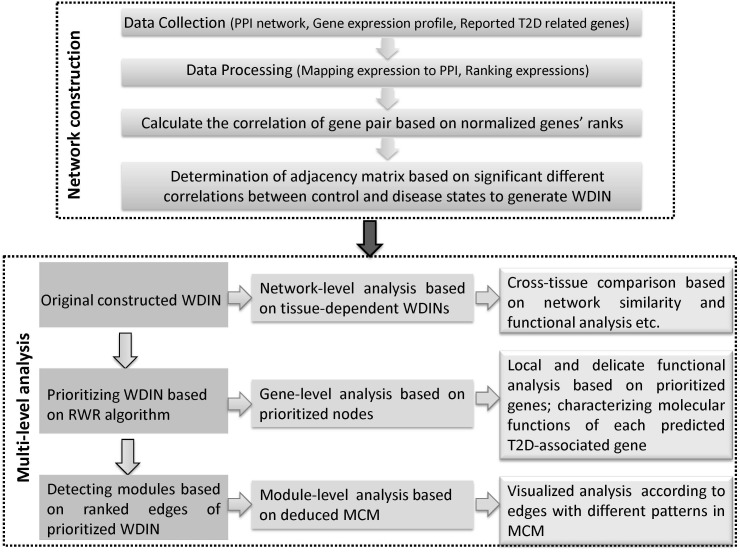
Schematic illustration of the multi-level comparative framework of this study. Two major phases are included, network construction and multi-level analysis. WDIN is the constructed disease network, and MCM is the identified key module.

Here, we propose a multi-level comparative framework mostly focusing on interactions across different representative tissues to gain a broader and further understanding of T2D. The whole framework can be divided into two phases and each phase is comprised of several major steps (Figure 1). The first phase is disease network construction, to effectively characterize the abnormal information response to disease. In recent years, many efforts have been made to extract disease information through selecting co-expression gene pairs whose expressions were highly correlated across samples. These methods were under the hypothesis that genes associated with the same disorder tend to share common functional features, i.e., their protein products tend to interact with each other. However, genes show highly correlated patterns of expression in one biological state, but not in another, i.e., they may not be highly correlated across the entire dataset, and therefore they fail to be picked out by co-expression based methods (Zhang and Horvath, 2005; Fuente, 2010). For this reason, we proposed a method based on finding ‘diverse interactions’ according to the discrepancy between correlations in different phenotypes by extending our previous work (Sun et al., 2013). Beyond our previous work, we further considered the weight of interactions together, thus all the diverse interactions along with their discrepant coefficients which composed the weighted diverse interaction network (WDIN). Indeed, this WDIN would unravel the complexity of gene-pair regulation in the complex process regarding different tissues. In addition, it should be noted that during the computation of adjacency matrix for WDIN, we proposed to calculate the discrepant coefficient based on genes’ ranks instead of expression values. Such an operation would weaken the biased influence caused by different expression levels in different tissues and experiments and consequently provide a uniform scale for all samples independent of the dynamic range of a data profile (Le et al., 2010; Altschuler et al., 2013).

The second phase is multi-level analysis based on the inferred disease network. This phase is composed of network-, gene-, and module-level analysis. Network-level analysis gives an overall cross-tissue study about the T2D based on the constructed original tissue-dependent WDIN. Gene-level analysis is carried out through discussing the prioritized nodes of WDIN which can provide a local and delicate explanation for the disease. The major step of this phase is to discover core modules and then derive a merged core module. Following module-level analysis could carefully and visibly illustrate the common and specific features belonging to different tissues. In this part, the random walk restart (RWR) algorithm is employed to rank genes and further extended to prioritize gene interactions. The clustering coefficient is introduced to enable identifying biological core modules composed of prioritized interactions.

In summary, we explore the tissue cross talk of T2D at gene-, module-, and network-level comparisons across different tissues to uncover hidden patterns and their biological implications from multi-tissues ‘omic’ data. Our multi-level comparative framework is shown in Figure 1. We expect that our proposed multi-level analysis framework can be extended beyond gene expression level and discover new commonalities and specificities among tissues.

## Materials and Methods

### Data Collection

#### Tissue Dependent Gene Expression Data Retrieval

We obtained gene expression profiles from three rat tissues (white adipose, skeletal muscle, and liver) [diabetes rats: Goto–Kakizaki (GK) rats; control rats: Wistar–Kyoto (WK) rats] from the National Center for Biotechnology Information (NCBI) Gene Expression Omnibus database (access ID: GSE13268, GSE13269, and GSE13270) (Almon et al., 2009; Nie et al., 2011; Xue et al., 2011). The profile was composed of 31,099 probes. Our first filter eliminated the probe sets without the corresponding official symbol, leaving 25,345 genes for further consideration.

#### T2D Associated Genes Collection

To provide support and verification for our work, we collected the canonically reported genes associated with T2D. These genes were gathered from Type II diabetes mellitus pathway (KEGG-Kyoto Encyclopedia of Genes and Genomes, H00409) which will be referred to as ‘T2D-pathway genes.’ In total, we obtained 50 T2D-pathway genes, among which 42 genes had gene expression aforementioned and were used in this study. Other T2D related genes were downloaded from the Rat Genome Database (RGD)^1^ in October 2018. In total, 515 genes were downloaded from RGD (referred to as RGD-reported genes), and only those that have been measured in GSE 13268-13270 were kept. Thus 303 RGD-reported genes were left.

#### Protein–Protein Interaction Network Integration

Rat protein–protein interaction (PPI) network was integrated based on KEGG pathway and BioGRID (Biological General Repository for Interaction Datasets). Actually, not all the proteins in PPI network have corresponding gene expression values in expression profiles GSE13268-GSE13270. To carry out our cross-tissue analysis, we only reserved interactions whose two nodes have expressions in three tissues. Finally, the reserved interactions composed a network with 4,081 nodes (proteins) among 24,503 edges (interactions). This network was noted as ‘background PPI network’ with gene pair-wise expressions in our study.

### Data Processing

In most studies about network biology such as the ones aimed at identifying network biomarkers of complex diseases, researchers usually collect gene expression data from multiple experiments. The expression profiles belonging to different experiments may represent different tissues, or different experiment conditions. Straightly pooling the expression profiles to construct various networks would ignore the underlying structure of the data, and the pooled estimates may be severely biased due to the heterogeneity of the experiments. Instead of pooling the expression data, we first ranked the genes by their expression values for each expression array, and then normalized the rank index for every gene. The normalized rank (z*-*score) was computed using the mean (μ) and standard deviation (σ) of the ranks of all genes along one sample which can be described as *z-*score = (x–μ)/σ. In subsequent computation and analysis, this normalized rank was used instead of expression value since it can reduce the data noise deriving from different experimental conditions and different tissues.

**FIGURE 2:**
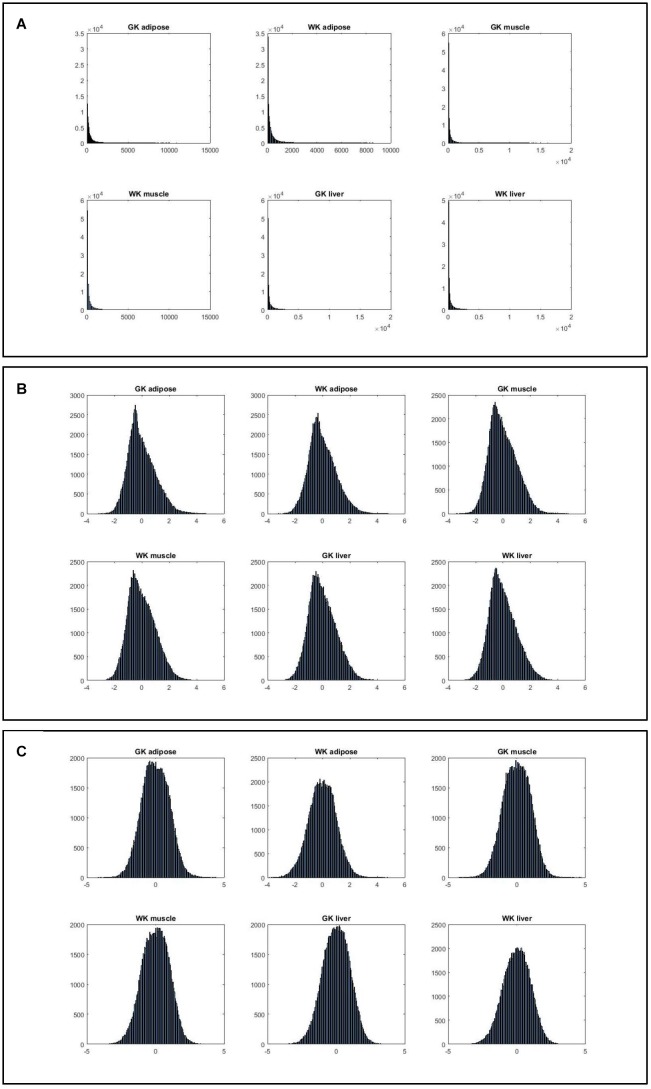
The histograms of six gene expression profiles under different data processing strategies. **(A)** Histograms of gene expression in three tissues for two phenotypes. **(B)** Histograms of normalized gene expression in three tissues for two phenotypes. **(C)** Histograms of gene index based on ranking expression in three tissues for two phenotypes.

Figure 2 shows that the expression levels estimated by normalized ranks subject to normal distribution are more consistent, indicating that such ranking processing is a more valid procedure.

### Tissue-Dependent WDINs Construction

A WDIN was constructed for each tissue that was related to the query disease—T2D. The pipeline of constructing WDIN was as follows:

(1) For each gene pair in the integrated background PPI network, calculate its correlation respectively under normal (WK) and disease condition (GK) based on the tissue specific normalized expression ranks of their corresponding two genes.
(2) Choose the gene pair as our diverse interaction whose correlations own significant difference between two conditions, and all identified dysfunctional interactions compose the WDIN. The weight of each edge is the difference between two correlations under normal (WK) and disease (GK) conditions.

After the creation of the tissue-dependent WDIN for each tissue, each interaction was weighted by the diverse correlation of corresponding gene pair. For each interaction, the absolute value of its weight can reflect the degree of deviation of this interaction in different phenotypes (normal and disease), and the positive and negative property of the weight coefficient indicates whether the corresponding function is active or inactive in disease condition compared with normal condition, which would provide more information for medical biology. Subsequently, we noted the edge with positive weight as ‘active edge,’ and the edge with negative weight as ‘inactive edge,’ respectively.

### Random Walk With Restart (RWR) on WDIN

#### Rank Candidate Genes

We applied RWR algorithm to our constructed tissue-dependent WDINs. The goal is to rank genes in candidate sets based on their association level with T2D. RWR is a ranking algorithm which simulates a random walk on the network to compute the proximity between two nodes by exploiting the global structure of the network. It starts on a set of seed nodes, which is the set of genes known to be associated with a phenotype p (T2D in this work). The candidate genes are then ranked by the probability of the random walker reaching this node (Lovasz, 1996; Tong et al., 2008; Ganegoda et al., 2014).

Each tissue-specific WDIN can be mathematically described as Γ = (*V*, ε, *w*), *V* is the gene set of WDIN’s nodes, εis a set of undirected interactions between these genes (or their products), *uv* ∈ ε represents an interaction between *u* ∈ *V* and *v* ∈ *V*, *w*(*u*, *v*) indicates the weight coefficient of interaction *uv* ∈ ε. The set of interacting partners of a gene *v* ∈ *V* is defined as *N*(*v*) = {*u* ∈ *V* } : *uv* ∈ ε and the total reliability of known interactions of *v* is defined as *W*(*v*) = ∑_u∈ N(v)_ *w*(*uv*).

Let *p*_0_ be the initial probability vector and *p*_s_ be a vector in which the i-th element holds the probability of finding the random walker at node *i* at step*s*. Algorithmically, random-walk based association scores can be computed iteratively as follows:

(1) $$p_{\text{S}+1}=(1-\text{γ})M^{\text{T}}p_{\text{s}}+\text{γ}(1-\text{η})p_{0}$$

Here, η denotes the weight of the network. γ ∈ (0,1)is a user-defined restart probability to adjust the preference between the importance of a protein or gene with respect to the seed set and network topology. Numerical results show that γ = 0.3 is optimal for RWR’s performance (Erten, 2009; Erten et al., 2011). Thus, γ is set to 0.3 for RWR in this paper. *M* is the transition matrix of the Γ, the transition probability from gene *u* to gene *v* can be described as follows,

(2) $$M(u,\;v)=\{\begin{array}{ll} w(u,\;v)/W(v), & \mathrm{if uv}\in \varepsilon \\ 0, & \mathrm{otherwise} \end{array}$$

The seed set is composed of T2D-pathway genes covered by the WDIN, and the candidate set contains other nodes of WDIN excluding these seed genes. The RGD-reported genes would be used as test genes to verify the performance of applying RWR to WDINs. The details will be shown in the Results section.

#### Rank Candidate Edges

Based on the ranked candidate nodes, we further prioritized the edges of WDIN for each tissue. In detail, given an edge, we assigned an index to the edge according to the ranks of its two linked nodes to indicate its association degree to the studied disease. Theoretically and computationally, using the average ranks of its two nodes as the edge’s index would meet the requirement. Then all the edges would be ranked in ascending order according to their assigned indices. An edge having higher rank (with smaller rank value) is more closely associated with the studied disease. These sorted edges enable us to identify a minimal set composed of prioritized edges which can reflect plentiful information about the disease in the considered tissue. Such a set would be referred to as ‘core module’, which could provide some important information from another viewpoint.

### Network/Module Comparison

We quantified the similarity of two networks based on edges. Given two different networks, we supposed the numbers of edges belonging to these two networks to be respectively *N*_1_ and N_2_. Then we calculated the number of edges that are present in both networks (common edges) noted as variable *n*. We defined variable *x*_1_ as the ratio of the number of common edges (*n*) to the number of edges in the first network (*N*_1_) and defined variable *x*_2_ as the ratio of the number of common edges (*n*) to the number of edges in the second network (*N*_2_). Using variables *x*_1_ and*x*_2_, we introduced a *S-*score as the harmonic mean of *x*_1_ and *x*_2_ (Roy et al., 2013; Knaack et al., 2014). The formulae are as follows,

(3) $$\begin{array}{c} x_{1}=\frac{n}{N1}\\ x_{2}=\frac{n}{N2}\\ S-\mathrm{score}=\frac{1}{\frac{1}{2}\sum_{\text{i}=1}^{2}\frac{1}{x_{\text{i}}}} \end{array}$$

### Pathway and Motif Enrichment

Functional analyses about pathway and process enrichment have been carried out with the following ontology sources: GO Biological Processes, KEGG Pathway, Reactome Gene Sets. The analyses are performed through web tool Metascape^2^ (Tripathi et al., 2015).

## Results

### Network-Level Analysis Based on Tissue-Dependent WDINs

In order to better capture the disease related information, for each tissue, we have designed a new way to infer a tissue-dependent WDIN through exacting the interactions with significant diverse correlations in different phenotypes. Instead of choosing disease related genes individually, we picked out such genes in pairs. An edge would be picked out if its corresponding genes were strongly correlated (the Spearman correlation coefficient is larger than or equal to some threshold, such as 0.8) under one strain while not (e.g., less than 0.2) in the other condition. Such an edge implicated that the interaction between the genes was obviously perturbed under the disease and hence noted as ‘diverse interaction’ in this work. Subtracting the correlation coefficient in normal state from the coefficient in disease state, we have the weight of each diverse interaction.

#### Characteristics of WDINs

Through screening all edges in background PPI network, we identified 2,026/2,118/2,184 diverse edges among 1,710/1,783/1,793 nodes for adipose/muscle/liver respectively. We computationally validated these networks by examining the number of T2D-rpathway genes and RGD-reported genes covered by each tissue-dependent WDIN. We found that the created WDIN could hit most (exceed 83%) T2D-pathway genes and more than half RGD-reported genes (Table 1). This means that our designed way of constructing WDIN is effective, and through screening the background PPI network and cutting away those edges having loose or no association with the disease, the inferred WDINs could capture abundant disease related information with less edges.

**Table 1:** Characteristics of three tissue-dependent WDINs.

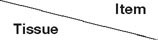	Number of nodes	Number of edges	Hits of 42 T2D-pathway genes	Hits of 303 RGD-reported genes
Adipose	2,026	1,710	35	154
Muscle	2,118	1,783	36	169
Liver	2,184	1,793	39	162

**FIGURE 3:**
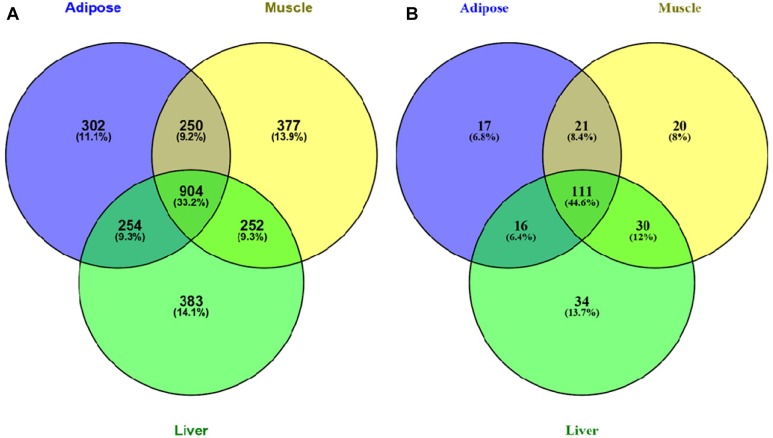
**(A)** Venn diagram of all genes in three tissue-dependent WDINs. **(B)** Venn diagram of T2D- related genes covered in three WDINs respectively.

We further investigated the Venn diagram for nodes of three tissue-dependent WDINs and disease related genes they covered (Figure 3). From the Venn we can see that the percentage of specific genes in each WDIN ranges from 11.1 to 14.1%, and the housekeeping genes (genes appeared in three WDINs simultaneously) is at the level of 33.2% (Eisenberg and Levanon, 2013; Lee et al., 2015). The Venn about the coved T2D-related genes composed of T2D-pathway genes and RGD-reported genes presented a similar result.

#### Network Similarity Analysis

**FIGURE 4:**
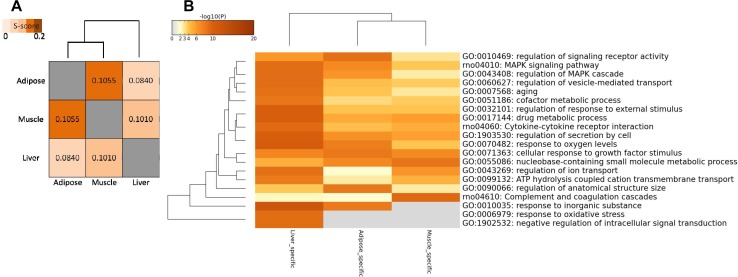
Network-level comparison of three tissue-dependent WDINs. **(A)** Network similarity based on edges. **(B)** Heatmap of enriched terms across three tissue-specific gene lists, colored by *p*-values.

In addition to comparing networks based on network nodes through Venn diagram, we also carried out network edge comparison to further quantify the extent of shared and tissue-specific network components. Thus, we introduced *S*-score measure to assess the similarity between networks edges for each pair of tissues since it is a more sensitive measure for comparisons. Based on *S*-score (Figure 4A), we found that the similarity between each pair of WDINs is low, which means that during the progress of T2D, three tissues possess evident tissue specificity. In this network comparison, adipose tissue and muscle tissue were indicated to emerge as the most similar dysfunctions caused by T2D among three insulin responsive tissues.

#### Cross-Tissue Functional Analysis Based on WDINS

To confirm the significant relation between tissue- dependent WDINs and T2D, the pathway and motif enrichment was conducted for each WDIN to categorize the genes participating in different biological functions or pathways.

##### Enrichment analysis on tissue non-specific genes

Table 2 lists the top 20 enriched terms of pathway and biological process of 904 tissue non-specific genes. The well-known T2D related pathway- Insulin signaling pathway ranks in the 16th position. Most other listed pathways have been documented to be associated with T2D. For example, two pathways about cancer (pathways in cancer and proteoglycans in cancer) were enriched, and this is not surprising since cancer is quickly emerging as another pathological consequence of T2D (Poloz and Stambolic, 2015). Due to the lack of adequate glucose uptake induced by dysfunction of insulin response, most pathways related to the cellular regulatory signal transduction of basic energy metabolism became abnormal, such as Chemokine, cAMP and Wnt signaling pathways and so on (Li et al., 2014). As T2D is a well-known metabolic disease, the metabolic related pathways appeared to be abnormal, such as Purine metabolism and drug metabolic process.

**Table 2:** Enriched terms of pathway and biological process of house-keeping genes.

Category	Description	Count	%	Log10(P)
KEGG Pathway	Pathways in cancer	174	19.3548	−100.0
KEGG Pathway	Chemokine signaling pathway	112	12.4582	−100.0
KEGG Pathway	Purine metabolism	104	11.5684	−91.7492
KEGG Pathway	Proteoglycans in cancer	101	11.2347	−79.4258
GO Biological Processes	Organophosphate biosynthetic process	139	15.4616	−70.6282
KEGG Pathway	Oxytocin signaling pathway	83	9.2324	−69.5144
KEGG Pathway	HTLV-I infection	105	11.6796	−65.5991
Reactome Gene Sets	Signaling by Receptor Tyrosine Kinases	105	11.6796	−64.0668
KEGG Pathway	cAMP signaling pathway	87	9.6774	−63.8028
GO Biological Processes	Small molecule biosynthetic process	143	15.9065	−59.8328
GO Biological Processes	Response to peptide	138	15.3503	−58.5452
GO Biological Processes	Drug metabolic process	148	16.4627	−55.7688
GO Biological Processes	Response to xenobiotic stimulus	108	12.0133	−54.4963
GO Biological Processes	Response to toxic substance	141	15.6840	−52.1495
KEGG Pathway	Insulin signaling pathway	67	7.4527	−52.0524
GO Biological Processes	Response to inorganic substance	143	15.9065	−51.8458
KEGG Pathway	Fluid shear stress and atherosclerosis	69	7.6751	−51.1151
KEGG Pathway	Leukocyte transendothelial migration	61	6.7853	−50.3888
KEGG Pathway	Wnt signaling pathway	66	7.3414	−49.2176
KEGG Pathway	Phosphatidylinositol signaling system	54	6.0066	−46.7635

##### Enrichment analysis on tissue specific genes

Some tissue-specific property can be displayed through the functional analysis of specific genes in three WDINs (Figure 4B). For example, Drug metabolic appeared to be significant in liver-dependent WDIN enriched terms. Negative regulation of intracellular signal transduction and response to oxidative stress were found to be dysfunctional in liver-specific WDIN only, and didn’t appear in the remaining two tissues. In addition, we found that overall the abnormal functions caused by T2D appear to be more similar in adipose and muscle, which is consistent with the result induced by network similarity based on *S*-score (Figure 4A).

### Gene-Level Analysis Based on Prioritized WDINs

#### Prioritizing WDINs Using RWR

To rank genes belonging to candidate sets based on their association level with T2D, we applied RWR to our three constructed tissue-dependent WDINs. The RWR method starts with the genes belonging to T2D pathway covered by each WDIN, and these genes are referred to as seed genes. The candidate set of genes includes other nodes of WDIN excluding the seed genes. After the random walking, the candidates are ranked according to the proximity of each gene to the genes in the seed set.

To verify the efficiency of random walk on the WDIN, we collected T2D related genes from RGD database. In all 303 RGD-reported genes appeared in our background network, and among 35 T2D-pathway genes hit in adipose-WDIN (seed genes), there are 0 overlaps. These 130 genes are pleasing test genes because we can verify our method through inspecting their rank indexes. Similarly, in muscle-WDIN, there are 36 genes from T2D-pathway (used as seed genes) and 169 RGD-reported genes respectively. In 169 RGD-reported genes, excluding the seed genes, there remains 146 genes which would be used as test genes for RWR. In liver-WDIN, there are 39 genes from T2D-pathway (used as seed genes) and 162 RGD-reported genes respectively. In 162 RGD-reported genes, excluding the overlap genes with seed genes, there remains 136 genes which would be used as test genes for RWR.

After random walking on the adipose-WDIN, the top 100 ranked candidates cover 16 test genes, top 200 covered 26, corresponding *p*-values tested by Fisher’s exact are 0.0019 and 0.0147. The corresponding results are listed in Table 3, along with the similar results for the other two tissues. This indicated that our random walking on inferred WDINs can effectively prioritized the disease related genes.

**Table 3:** We verify the efficiency of prioritized WDIN through Fisher’s exact test.

Tissue	Item	Top100	All candidates	Top200	All candidates
Adipose	Test gene covered	18	82	29	171
	Test gene uncovered	120	1593	109	1504
	Fisher’s exact	*p*-value = 0.0003		*p*-value = 0.0003	
Muscle	Test gene covered	16	84	25	175
	Test gene uncovered	138	1663	129	1572
	Fisher’s exact	*p*-value = 0.0069		*p*-value = 0.0198	
Liver	Test gene covered	13	87	23	177
	Test gene uncovered	131	1667	121	1577
	Fisher’s exact	*p*-value = 0.0492		*p*-value = 0.0336	
Adipose	Test gene covered	16	84	26	174
	Test gene uncovered	114	1591	104	1501
	Fisher’s exact	*p*-value = 0.0019		*p*-value = 0.0019	
Muscle	Test gene covered	14	86	22	178
	Test gene uncovered	132	1661	124	1569
	Fisher’s exact	*p*-value = 0.0310		*p*-value = 0.0490	
Liver	Test gene covered	11	89	21	179
	Test gene uncovered	125	1665	115	1575
	Fisher’s exact	*p*-value = 0.0989		*p*-value = 0.0435	

To collect more generally known T2D genes to verify our framework, we queried the approved type 2 diabetes genes through published papers from year 2010 to 2018. After mapping their homologous genes in rats based on the homologous categories from MGI (Bult et al., 2008), 12 genes were reserved. Finally, 8 of 12 genes were detected in three constructed WDINs which are respectively BCAR1, CAMK2B, CPS1, FADS2, GCK, PPARG, PDX1, and POLD2. Among them, GCK (Misra and Owen, 2018) and PDX1 (Kodama et al., 2016) were included in our seed set. Besides, FADS2 (Li et al., 2016) were ranked 112 in our prioritized adipose-WDIN (1,675 genes in total), and POLD2 (Gaudet et al., 2011) were ranked 98 in muscle-WDIN (1,747 genes in total). BCAR1 (Kazakova et al., 2018) and CAMK2B (Sacco et al., 2016) also have higher ranks in muscle-WDIN which were 289 and 257 respectively. PPARG (Voight et al., 2010) was ranked 533 in adipose-WDIN, while CPS1 (Matone et al., 2016) had an unsatisfied rank 1,272. In general, the ranked results of these 8 genes were basically consistent with their published results associated with T2D. Furthermore, our results could offer tissue-specific insights into T2D.

#### Cross-Tissue Functional Analysis Based on Prioritized Genes

**FIGURE 5:**
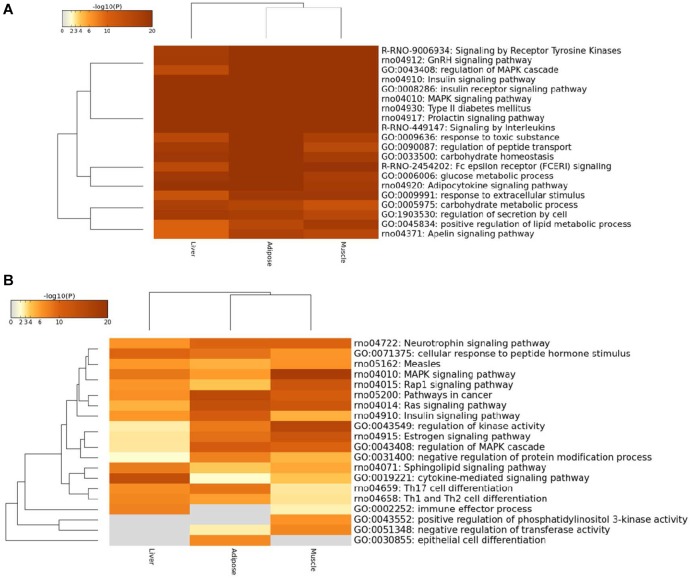
Heatmap of top100 genes cross three prioritized WDINs. **(A)** For tissue non-specific genes. **(B)** For tissue-specific genes.

In order to reduce the effect of ascertainment bias in genes loosely or less associated with the disease, we set a threshold, and only focused on the prioritized genes above this threshold in three tissue-dependent WDINs. Thus we can perform a local and more delicate functional analysis on tissue non-specific and tissue-specific genes among them separately with less noise. Our experiments show that the effect of the selection of the threshold is minor. If the threshold is too small such as less than 50, the genes chosen to perform functional enrichment analysis would be insufficient to achieve statistical significance; whereas if the threshold is too large such as higher than 200 (according to the results of the previous subsection), the follow-up functional analysis would be dilute on account of genes loosely associated with T2D. Therefore, we set a cutoff value for prioritized genes at 100 to conduct the cross-tissue functional analysis, and the results were shown in Figure 5.

We found that the enriched terms were more specific and striking with T2D when considering only the top100 prioritized housekeeping genes, such as type II diabetes mellitus, insulin signaling pathway, and glucose metabolic process. When restricting tissue-specific genes in the top100 prioritized genes, the tissue specificity became more visible. In detail, the immune effector process and positive regulation of phosphatidylinositol 3-kinase activity were not enriched in adipose, while specific genes in liver were not involved in negative regulation of transferase activity, positive regulation of phosphatidylinositol 3-kinase activity and epithelial cell differentiation process which also means that only adipose takes part in epithelial cell differentiation. When compared with the other two tissues, the muscle displays significant enrichment in regulation of kinase activity process and MAPK signaling pathway. And also from the overall view, the enriched functional items for adipose and muscle were closer.

#### Characterizing the Molecular Functions of Each Predicted T2D-Associated Gene

After ranking candidates through random walking on tissue-dependent WDINs, we found that some genes were ranked ahead while they did not appear in the RGD database or T2D-pathway. For clarity, we focused on top50 nodes and considered them as potential T2D-associated genes. Below, we respectively list their gene information in Table 4, and corresponding pathway and process enrichment in Table 5.

**Table 4:** Annotation on predicted potential T2D-assocaited genes.

Tissue	Gene symbol	Rank index	Description
Adipose	CARD9	2	Caspase recruitment domain family, member 9
	CHUK	9	Conserved helix-loop-helix ubiquitous kinase
	FBP1	6	Fructose-bisphosphatase 1
	FOS	7	Fos proto-oncogene, AP-1 transcription factor subunit
	GNB4	15	G protein subunit beta 4
	NFKBIB	4	NFKB inhibitor beta
	PGM1	3	Phosphoglucomutase 1
	PRPS1	12	Phosphoribosyl pyrophosphate synthetase 1
	RXRA	5	Retinoid X receptor alpha
	SHC1	10	SHC adaptor protein 1
	STAT4	1	Signal transducer and activator of transcription 4
Muscle	ATP5O	12	ATP synthase peripheral stalk subunit OSCP
	CAMK2A	6	Calcium/calmodulin-dependent protein kinase II alpha
	GLB1	3	Galactosidase, beta 1
	MAP3K7	4	Mitogen activated protein kinase kinase kinase 7
	PDGFRA	11	Platelet derived growth factor receptor alpha
	PRKACB	5	Protein kinase cAMP-activated catalytic subunit beta
	RASGRF1	9	RAS protein-specific guanine nucleotide-releasing factor 1
	RPS6KA1	2	Ribosomal protein S6 kinase A1
	STAT4	7	Signal transducer and activator of transcription 4
liver	CNTFR	5	Ciliary neurotrophic factor receptor
	GALM	8	Galactose mutarotase
	MAP2K7	9	Mitogen activated protein kinase kinase 7
	PSMD9	3	Proteasome 26S subunit, non-ATPase 9
	PTPN6	6	Protein tyrosine phosphatase, non-receptor type 6
	RASGRF1	1	RAS protein-specific guanine nucleotide-releasing factor 1
	RASGRF2	7	RAS protein-specific guanine nucleotide-releasing factor 2
	RASGRP2	10	RAS guanyl releasing protein 2
	RYR3	4	Ryanodine receptor 3

**Table 5:** Key issues of motif enrichment analysis on predicted potential T2D-assocaited genes.

Tissue	Category	Description	Log10 (P)	Hit genes
Adipose	Reactome Gene Sets	Signaling by Interleukins	−6.77	Nfkbib, Shc1, Chuk, Fos, Stat4
	KEGG Pathway	Th1 and Th2 cell differentiation	−6.64	Nfkbib, Chuk, Fos, Stat4
	KEGG Pathway	Pentose phosphate pathway	−6.12	Fbp1, Pgm1, Prps1
	Reactome Gene Sets	Innate Immune System	−5.75	Pgm1, Card9, Nfkbib, Shc1, Chuk, Fos
	KEGG Pathway	Chemokine signaling pathway	−5.57	Nfkbib, Shc1, Gnb4, Chuk
	Reactome Gene Sets	Fc epsilon receptor signaling	−4.63	Shc1, Chuk, Fos
	GO Biological Processes	Cellular response to insulin stimulus	−3.47	Fbp1, Rxra, Shc1
Muscle	KEGG Pathway	MAPK signaling pathway	−7.15	Pdgfra, Rps6ka1, Rasgrf1, Prkacb, Map3k7
	KEGG Pathway	Long-term potentiation	−5.41	Camk2a, Rps6ka1, Prkacb
	KEGG Pathway	Wnt signaling pathway	−4.38	Camk2a, Prkacb, Map3k7
	KEGG Pathway	Calcium signaling pathway	−4.04	Pdgfra, Camk2a, Prkacb
Liver	KEGG Pathway	MAPK signaling pathway	−5.30	Rasgrf2, Rasgrf1, Rasgrp2, Map2k7
	GO Biological Processes	Regulation of cation transmembrane transport	−3.31	Rasgrf2, Ptpn6, Rasgrf1

According to the annotation of genes by Metascape, we found that some detected genes indeed have close connection with T2D. Among the top50 potential genes in adipose-WDIN, PGM1 mainly takes part in Pentose phosphate pathway and innate immune system and galactose catabolic process. FBP1 responses to insulin stimulus and pentose phosphate pathway. STAT4 was ranked in the top50 in both prioritized adipose- and muscle- WDINs respectively. This gene provides instructions for a protein that acts as a transcription factor, which means that it attaches (binds) to specific regions of DNA and helps control the activity of certain genes. The STAT4 protein is turned on (activated) by immune system proteins called cytokines, which are part of the inflammatory response to fight infection. RASGRF1 appears in potential T2D-associated gene set in both prioritized muscle- and liver- WDINs, and it has been shown to be upstream from IGF1 (Insulin-like growth factor 1) which is a star gene about T2D, allowing it to control growth in mice (Drake et al., 2009). PSMD9 was observed in top50 genes detected in liver-WDIN, and it plays an important role in negative regulation of insulin secretion processes (GO:0046676) and positive regulation of insulin secretion (GO:0032024); GALM is another potential T2D-related gene detected in liver-WDIN that acts as a part of the galactose catabolic process (GO:0019388) and the galactose metabolic process (GO:0006012).

These potential T2D-associated genes would be helpful for physicians or biologists, as they can be used to determine an experimental target as the subject of future research.

### Module-Level Analysis for the Predicted T2D Associated Genes

#### Identifying Core Module From Prioritized WDIN Based on Edges

In addition to prioritizing candidate nodes, we further ranked interactions in three WDINs separately to capture those crucial interactions in several disorders caused by T2D. Specifically, each edge of the WDIN would be ranked according to the average rank of its corresponding two nodes. Theoretically, the edge having higher ranks (with smaller rank values) tends to have a closer association with the studied disease.

Our final goal was to identify a minimal set of prioritized WDIN edges which can reflect copious information about the disease in some tissues, namely ‘core module’ hereafter. To achieve this goal, we carried out a series of tests to evaluate the effectiveness of predicting and detecting disease related genes for the subnetworks from prioritized tissue-specific WDIN. Firstly, for each tissue, the series subnetworks were successively exacted from the prioritized WDIN based on edges from upon top 2.5% to upon top 25% at an internal of 2.5, and then the maximum connected component (MCC) of each subnetwork was retained for further analysis. In total, we had 10 MCCs for each tissue. Secondly, we compute the numbers and the coverage rates of the disease related genes collected from T2D-pathway and RGD database hit by each MCC (Supplementary Figure S1).

We then identified our core module through investigating the clustering coefficient of each MCC because the clustering coefficient can reflect the modular feature of biological function module (Caroline and Ralf, 2006). In most cases, a complex with larger clustering coefficient frequently tends to form a biological functional module. Generally, the clustering coefficient ranges from 0 to 1, and for a stochastic network with *N* nodes the value of it is approximately equal to *N*^−1^.

**FIGURE 6:**
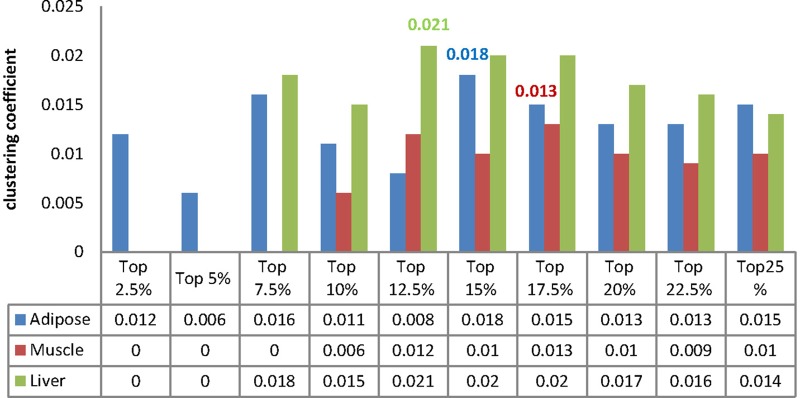
Clustering coefficient of MCCs for each tissue.

We calculated the clustering coefficient for each MCC and the corresponding results were listed in Figure 6. Besides, we compute the clustering coefficients of three tissue-dependent WDINs which are 0.011, 0.011, and 0.016 for adipose, muscle, and liver respectively. For each tissue, the MCC with the largest clustering coefficient was selected as our core module, that is to say, top 15% of adipose-WDIN, top 17.5% of muscle-WDIN and top 12.5% of liver-WDIN are taken as our tissue-dependent core modules. It should be noted that the clustering coefficients (0.018, 0.013, and 0.021) belonging to three identified modules are all larger than that of their corresponding WDINs.

Analysis of core modules and a focus on the difference between the edges/interactions instead of nodes/genes individually would provide some important information from another viewpoint. We will illustrate this point in the next subsection.

#### Creating Merged Core Module (MCM) Through Jaccard Index

Here we created the merged core module (MCM) by integrating three tissue-dependent core modules through introducing ‘Jaccard index’ (Knaack et al., 2014). For each pair of core modules, we calculated the Jaccard index, which for a pair of sets is defined as the ratio between the size of the intersection and the size of the union of two sets. If the Jaccard index of an interaction is higher than a threshold in at least one pair of modules, we added this interaction to the merged core module along with their weights. To find a suitable threshold, we separately calculated a series of clustering coefficients of complexes which were generated when the threshold was set as 0.3, 0.2, 0.1, and 0.05, and the resulting clustering coefficients were 0, 0, 0.027, and 0.026. Hence, the parameter α = 0.1 was finalized as the best threshold to create our MCM. Finally, we had an MCM composed of 198 edges among 154 nodes.

We then investigated the inferred MCM to identify the specific and common components of dysfunctions between different tissues.

#### Cross-Tissue Analysis Based on Merged Core Module

**FIGURE 7:**
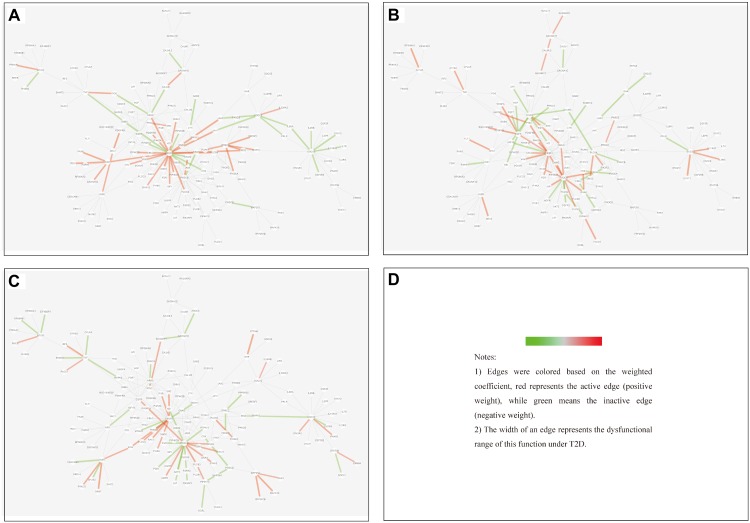
Tissue-specific edges displayed in MCM. **(A)** Adipose. **(B)** Muscle. **(C)** Liver.
(2) Differential edge: the edge was significantly dysfunctional in at least two tissues. While their statuses were different, for example, the edge was active in tissue A whereas inactive in tissue B (shown in Figure 8).

To systematically assess the extent to which the dysfunctions were shared among different tissues, we split the edges of MCM into three categories:

(1) Tissue-specific edge: the edge was significantly dysfunctional in only one tissue (as shown in Figure 7).

**FIGURE 8:**
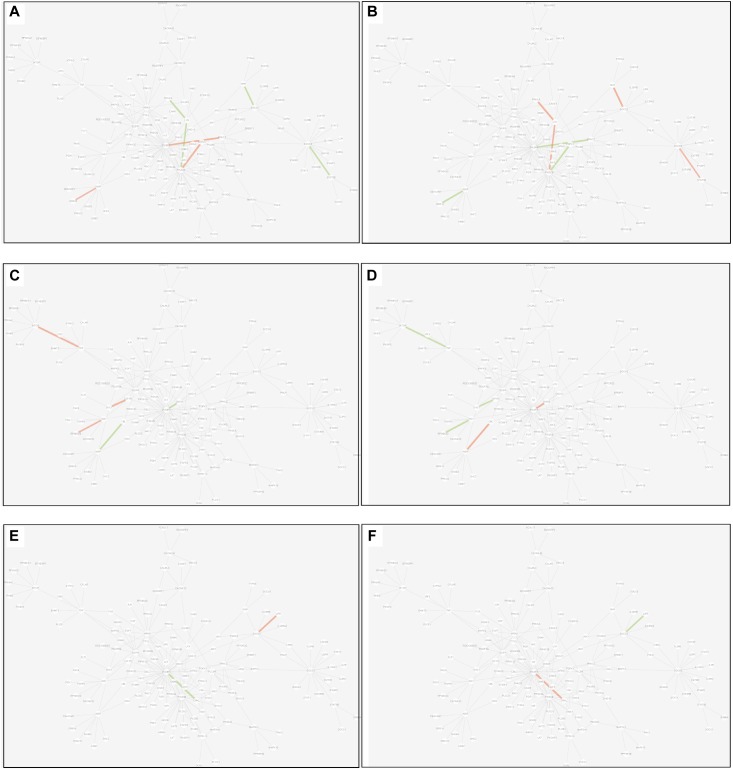
Differential edges displayed in MCM. The two subgraphs in each row show the differential edges between tissue I and tissue II. The different status between the tissue pair under T2D can be exhibited through the dysfunctional weight of the edge in different tissue-dependent WDIN. **(A,B)** Show the differential edges between adipose and muscle, respectively. **(C,D)** Show muscle and liver, respectively. **(E,F)** Show adipose and liver, respectively. The color and width of the edge here have the same meaning as in Figure 7.
(3) Common edge: the edge was significantly dysfunctional in at least two tissues. When the status was consistent, for example, the edge was active in tissue A whereas inactive in tissue B (shown in Figure 9).

**FIGURE 9:**
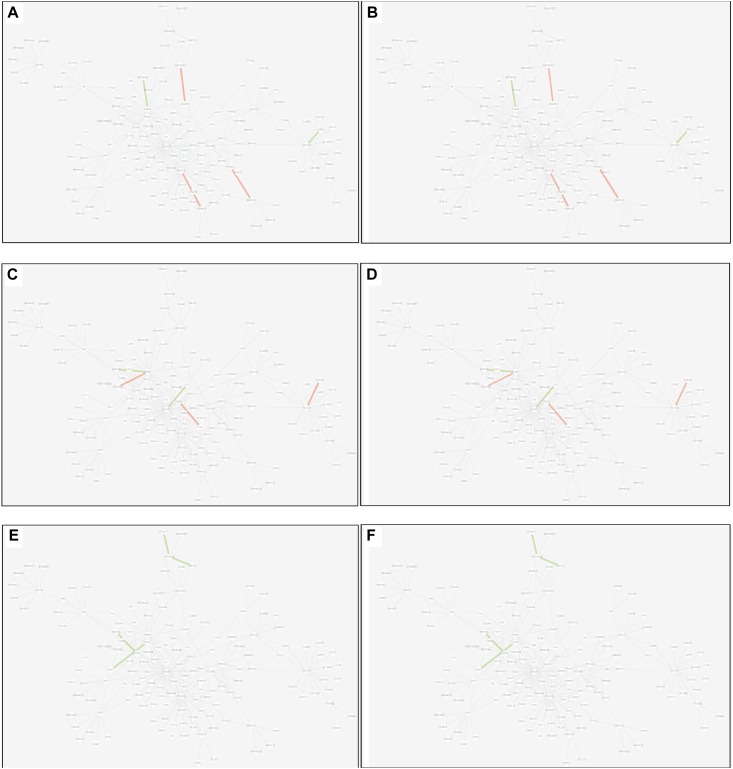
Common edges displayed in MCM. The two subgraphs in each row show the common edges between two tissues. The consistent status between the tissue pair under T2D can be exhibited through the dysfunctional weight of the edge in different tissue-dependent WDIN. **(A,B)** Are the common edges between adipose and muscle, respectively. **(C,D)** Are for muscle and liver, respectively. **(E,F)** Show adipose and liver, respectively. The color and width of the edge here have the same meaning as in Figure 7.

We found that in MCM, most adipose-specific edges appeared in links to hub gene PIK3R1, and a large proportion of them were active under T2D, such as the function occurred between PIK3R1 and INS1, the function between PIK3R1 and IRS2, the function between PIK3R1 and IGF1 etc. Taking muscle-specific edges in MCM into consideration, it can be seen that PIK3R1, PIK3CB, and EGFR were key nodes. The corresponding functions linked to PIK3R1 (such as the function occurred between PIK3R1 and IGF1R) were inactive which made the condition different than in adipose, and the corresponding functions around EGFR were active (such as functions linked between this node and IGF1). Though the distribution of liver-specific edges in MCM was decentralized when compared with other two tissues, PIK3R1 and PIK3CB were still two key nodes, interactions between PIK3R1 and other nodes (such as INS2) were active, while PIK3CB was a balanced node in terms of the hallmark of interactions.

There were 7 differential edges between adipose and muscle in MCM, which means the corresponding functions presented opposite status in these two tissues. As documented (Bereziat et al., 2002), GRB14 inhibited the catalytic activity of the INSR, which was identical to our result displayed in Figure 8B. Actually, according to our result, this inhibition may have only emerged in muscle tissue, while it would be inverted in adipose tissue (Figure 8A) This provides a meaningful target for biological experiments.

All 5 differential edges were identified between muscle and liver in MCM (Figure 8C,D). Also from the visible changes in corresponding functions we could reveal more subtle and interesting difference between tissues. For example, as described in GeneCards^3^, SHC1 interacts with the NPXY motif of tyrosine-phosphorylated IGF1R. According to our results, we can further infer that the interaction was active in muscle under T2D while inhibited in liver. Some similar results would be observed between adipose and liver.

Inspecting the common edges between tissue pair, a notable thing was that all the four common interactions between adipose and liver were inactive. Remarkably, one of these four edges was between ABCC8 and KCNJ11, which are two well-known T2D-related genes encoding proteins Kir6.2 and Sur1, respectively, in pancreatic beta cells. KCNJ11 interacts with ABCC8 to produce the KATP channel, which transfers potassium ions across the beta cells (Haghvirdizadeh et al., 2015). This interaction was indirectly linked through CACNA1E and was inhibited in both adipose and liver.

However, here we only described several edges for each category. Similar results can be examined from Figure 6–9 which would be useful in studies about disease mechanism through analyzing the shared and specific components among tissues under the disease.

## Discussion

Type 2 diabetes (T2D) is a complex disease and its dysfunction involves many tissues. This work systematically investigates commonalities and specificities of T2D among multiple tissues. We established a multi-level comparative framework across three insulin target tissues (white adipose, skeletal muscle, and liver) to provide a better understanding of T2D.

The first challenge is to represent the tissues from the data. Starting from the ranks of gene expression, we constructed the ‘disease network’ through detecting diverse interactions to provide a well-characterization for disease affected tissues. Based on the constructed tissue-dependent WDINs, an elementary and integral comparative analysis at network-level was conducted. The results of network similarity according to the edges of network indicated that the similarity among three tissues is lower, thus justifying the necessity to conduct tissue-specific analysis for T2D. The differences among tissues were also visible in enriched motif based on tissue-specific genes, and these differences showed that some T2D-related pathways or biological processes own tissue specificity. Besides, we found that among three tissues, adipose and muscle have more similar components in terms of both enriched functions and network similarity.

To reduce the negative effects induced by genes loosely associated with T2D, RWRs algorithm was applied to the disease network to prioritize its nodes and edges according to their associations with T2D. Genes ranked higher theoretically are significantly associated with T2D. Gene-level analysis was carried out on those genes ranked higher such as in the top100. On one side, we discussed these genes individually and found that some of them have been reported to be related with disease genes, while several are not yet documented and could be potential T2D-related genes which may be further verified experimentally. On the other side, we collected these genes together to survey their combined functions through inspecting the enriched pathways and biological processes. Compared with the similar analysis based on the whole disease network, the analysis based on those closely associated with T2D displayed more specific and striking enriched issues with T2D (such as type II diabetes mellitus, insulin signaling pathway, and glucose metabolic process).

The network- and gene- level analyses could give some novel information cross tissues; meanwhile, they largely verified the effectiveness of our random walking on the constructed WDINs. Thus, based on the prioritized edges of WDINs, we further identified a merged core module (MCM) by combining the clustering coefficient and Jaccard index, which can provide and elaborate and visible explanation about the common and specific features for different tissues at module- level. Edges in the MCM were grouped into three categories: specific edges, differential edges, and common edges. Focusing on these three categories of edges, more detailed common issues and specific differences of dysfunctional functions between tissues were revealed which would enable us to further understand the disease. We await the emergence of tissue-specific and isoform-specific gene (specifically genes from IRs) knockout studies to corroborate these conclusions.

Overall, we presented a mathematical and systems biology framework consisting of constructing tissue-dependent disease related networks and multi-level analysis based on these constructed networks. Our network-, gene-, and module-level characterization across different tissues of T2D hold the promise to provide a broader and deeper understanding for T2D mechanism.

## Data Availability

Publicly available datasets were analyzed in this study. This data can be found here: www.ncbi.nlm.nih.gov/geo.

## Author Contributions

YW and SS designed the study. FS organized the database and analyzed the results. SS and YW wrote the manuscript. All authors read and approved the submitted version.

## Conflict of Interest Statement

The authors declare that the research was conducted in the absence of any commercial or financial relationships that could be construed as a potential conflict of interest.

## Footnotes

1 http://rgd.mcw.edu/wg/home

2 http://metascape.org

3 https://www.genecards.org
