# Supplementary material for: Multi-Level Comparative Framework Based on Gene Pair-Wise Expression Across Three Insulin Target Tissues for Type 2 Diabetes
Source: Front Genet. 2019 Mar 26;10:252. doi: 10.3389/fgene.2019.00252 (PMC6443994; doi:10.3389/fgene.2019.00252)
Supplement: Supplementary file 1 [file Image_1.pdf]

Supplementary Material

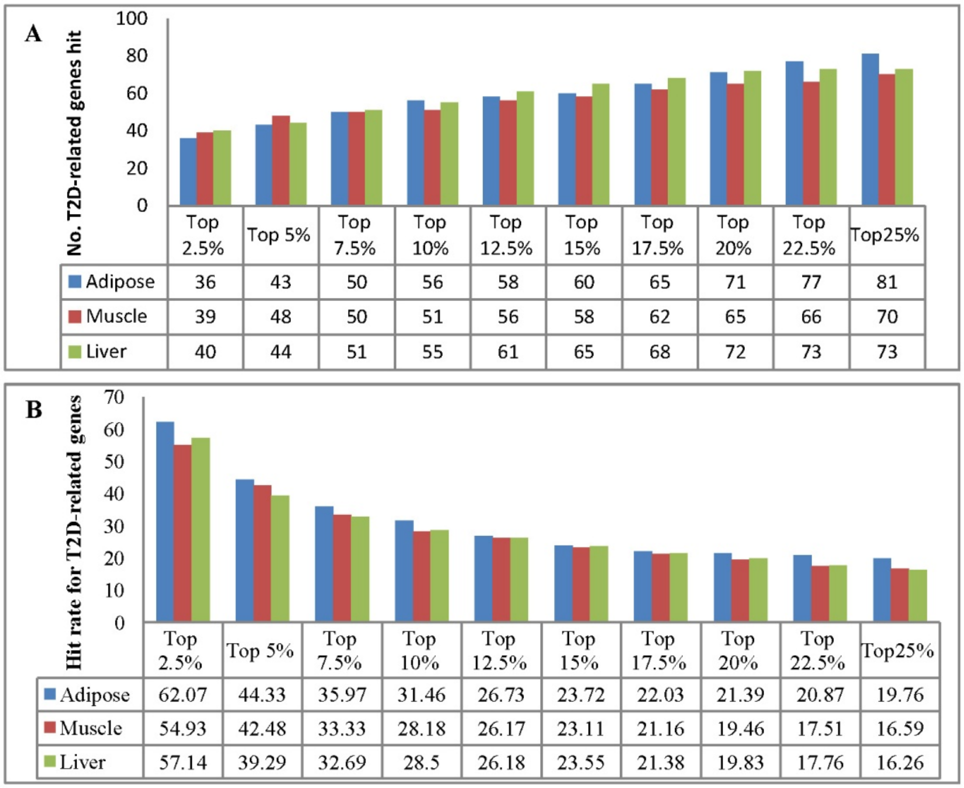

	Top 2.5%	Top 5%	Top 7.5%	Top 10%	Top 12.5%	Top 15%	Top 17.5%	Top 20%	Top 22.5%	Top25%
Adipose	36	43	50	56	58	60	65	71	77	81
Muscle	39	48	50	51	56	58	62	65	66	70
Liver	40	44	51	55	61	65	68	72	73	73

	Top 2.5%	Top 5%	Top 7.5%	Top 10%	Top 12.5%	Top 15%	Top 17.5%	Top 20%	Top 22.5%	Top25%
Adipose	62.07	44.33	35.97	31.46	26.73	23.72	22.03	21.39	20.87	19.76
Muscle	54.93	42.48	33.33	28.18	26.17	23.11	21.16	19.46	17.51	16.59
Liver	57.14	39.29	32.69	28.5	26.18	23.55	21.38	19.83	17.76	16.26

Figure S 1. The numbers **(A)** and the coverage rates **(B)** of the disease related genes collected from T2D-pathway and RGD database hit by each MCC.
